# Nowhere land: The evicted space of Black tenants’ rights in Montreal

**DOI:** 10.1177/02637758211041120

**Published:** 2021-09-02

**Authors:** Ted Rutland

**Affiliations:** Concordia University, Canada

**Keywords:** Property, housing, tenants’ rights, race, blackness, Montreal

## Abstract

Property relations in 1980s Montreal were a venue of struggle and change. In this period, a well-organized tenants’ movement and the election of progressive governments spawned a series of legal and policy changes that strengthened tenants’ rights in the city. During the same period, however, an emerging police, government and media discourse cast Black communities as criminal ‘ghettos’, and a variety of mechanisms, including new policies meant to protect tenants’ rights, were used to evict criminalized Black tenants. Guided by recent work on property and Black geographies, respectively, this article examines how racial subjects are constituted in struggles over tenants’ rights. The racial limits of tenants’ rights in Montreal, it argues, are traceable to the socio-spatial relations of slavery and the intensifying criminalization of Black life in the 1980s, each of which nullified Black spatial belonging in the city. The tenant, the article concludes, is never just a tenant, but also a racial subject – a subject formed at the edges of blackness. In a terrain forged by slavery and its afterlives, the possibility of expansive tenants’ rights presupposes a right systemically denied in advance for Black people in the Americas: the right to exist here in the first place.

Property relations in 1980s Montreal were a venue of struggle and change. In this period, a well-organized tenants’ movement and the election of progressive governments at the provincial and municipal levels spawned a series of legal and policy changes. These changes sought to improve tenants’ living conditions and protect them from displacement through a new ‘right to remain’ in their homes. This new right, though it could be abrogated by landlords under certain conditions, gave tenants a more advantageous position in what Blomley (2020) calls ‘the meshwork of property’. During the same period, however, an emerging discourse cast racialized and particularly Black spaces as interruptions in the urban fabric, urban ‘ghettos’ where prevailing social and spatial norms were overturned. These discourses were joined, in 1989, by an intensified police campaign against supposed Black drug dealers in ‘ghetto’ spaces. The city government, in addition to supporting the police campaign, employed new policies and programs to evict and otherwise displace criminalized Black tenants, an action seemingly anathema to the government’s commitment to tenants and their newly recognized right to remain.

Property relations and race have long been recognized as interconnected, perhaps especially in settler-colonial contexts like Montreal. In such contexts, Indigenous dispossession was the precondition for property in land, while enslaved Africans were reduced to the status of moveable property (Arneil, 1996; Harris, 1993). The point, as scholars increasingly emphasize, is not simply that property is codified in racially exclusive terms but that property discourses and relations help to define racial ontologies. As Bhandar (2018: 8) explains, ‘prevailing ideas about racial superiority’ have shaped who is and is not permitted to own property, while the concept of race ‘was and remains subtended by property logics’ in turn. Reworking a concept from Cedric Robinson, Bhandar argues that property relations are ‘racial regimes of ownership’ that structure access to land and other entities alongside and through the making of racial hierarchies. What happens, however, when we look beyond property *ownership*? What happens when another social class – tenants – struggle to improve their social position within property relations? Are ‘prevailing ideas about racial superiority’ subverted in these political developments? Or do racial ideas instead inform, and result from, important divisions within the social group whose rights suddenly gain new recognition and protection?

This article pursues these questions, examining how racial subjects are formed and deformed in struggles over tenants’ rights. It focuses in particular on political changes in 1980s Montreal, including the recognition of a new tenant right – ‘the right to remain’ – and new housing policies meant to improve tenants’ living conditions and protect their rights. These developments, while beneficial to many tenants, were shaped by new and longstanding ideas about Black spatiality. These ideas, I suggest, emerged in the thought of French jurists, who provided a justification for slavery in the Americas that foreclosed the possibility of Black citizenship, territorial belonging or property ownership. Centuries later, a variation on these ideas, now more closely linked to the criminalization than enslavement, shaped the struggle for tenants’ rights in Montreal. The result was a set of housing institutions, policies and programs that provided new protections for ‘tenants’ alongside and through the displacement of Black tenants. The tenant, I conclude, is never just a tenant, but also a racial subject – a subject formed at the edges of blackness. In a terrain forged by slavery and its afterlives, the possibility of expansive tenants’ rights, including the ‘right to remain’, presupposes a right systemically denied in advance for Black people in the Americas: the right to exist here in the first place.

## Blackness, territory and property

My entry point to the entanglements of property and racial subjects is provided by Black scholars who have examined the racial limits of modern (European) conceptions of the human being. This line of work, often traced back to Fanon (1952), reveals how new conceptions of the human were developed as Europeans built extensive regimes of slavery, colonialism, plunder and genocide. It was in this context, Wynter (2003) argues, that the human being appeared in European thought for the first time, not as a creature of God, but as the juridico-political and eventually biopolitical subject of the state. Displaced from these conceptions were the populations contemporaneously enslaved and/or colonized by European states, companies and citizens, an ontological displacement that was premised on the claim that the subjugated populations lacked the essential characteristics of the human (e.g. sentience, rationality). The modern meaning of ‘race’ emerged in this tense, recursive relationship between what Purifoy (2018: 15) terms ‘abstract ideas’ and ‘socio-legal institutions’. An African was racialized as Black, as Vergès (2006) explains, ‘the moment they were sold as a slave’. Once racialized as Black, moreover, a person was excluded from ‘humanity and the category of the free’ (23–24).^1^ Modern conceptions of the human, therefore, were produced alongside and through ‘a refusal of black humanity and a refusal to recognize the *struggle* to assert black humanity’ (McKittrick, 2017: 98).

These insights make it possible to analyse contemporary social relations. The abolition of slavery, after all, did not abolish these anti-Black conceptions of the ‘human’ or associated violences. The latter, instead, have remained entangled with an array of new and preserved socio-legal institutions into the present. Central to this article is the post-slavery articulation of blackness and criminality. A substantial literature examines this articulation in the United States, showing how blackness passed from a signifier of slavery to a signifier of criminality and came to anchor an array of new, repurposed and ever-evolving practices of surveillance, captivity and premature death (cf. Davis, 1997, James, 2005). A small but growing literature traces this dynamic elsewhere in the Americas, including Latin America (Alves, 2018, 2019; Da Silva, 2014), the Caribbean (Paton, 2004) and Canada. In Canada, Maynard (2017: 32) shows how a set of meanings attached to blackness under slavery, including criminality, ‘created a road map for [the] treatment of Black life throughout the nineteenth and twentieth centuries’. Many writers and activists have also shown how a particular criminal category, ‘the gang’, has functioned in Eastern Canada as ‘code for Black people’ since the 1980s (Cole, 2020; Hudson and Loreto, 2021; Livingstone, 2018; Maynard, 2017).

Anti-Black ontologies, whether during the era of slavery or its afterlives, are tied to geography in complex ways. At a broad scale, Bledsoe (2015: 324) argues that ‘the supposed non-being of the Black [person]’ is tied to ‘the assumed inability of Black space to exist’. Black people are thus imagined by non-Black people to be ‘ungeographic’ (McKittrick, 2006: x). This imaginative ungeography takes different forms in different national and sub-national contexts. In many contexts, including Canada and Europe, blackness operates largely as ‘a symbol of [national] non-belonging’ (Hawthorne, 2017: 160). As Walcott (1997: 43) argues, the usual assumption in Canada is that ‘any Black presence’ is a ‘recent and urban one’ spawned by migration, effacing centuries of Black history. In Quebec, a Canadian province with its own national identity, Mugabo (2016b: 168) argues that blackness and Black suffering are cast ‘outside the national narrative’, making Black life a conceptual ‘impossibility’. At smaller scales, the assumed ‘nowhereness’ of Black life is inscribed in an array of institutions and practices that displace and/or confine Black people – two ways in which they are denied a ‘proper place’ (Shabazz, 2015: 24). These include the practices of Jim Crow (Harris and Hyden, 2018; Woods, 1998), policing (Alves, 2018; Maynard, 2017), prisons (Gilmore, 2007; Shabazz, 2015) and urban planning (Allen, 2019; Bates, 2006; Brand, 2018; Rutland, 2018.; Simone, 2016)

Property relations are a particular way of conjuring racial subjects and racialized spaces. Slavery, as I noted, reduced captured Africans to the status of moveable property while barring them from acquiring property of any kind. As Hong (2020) explains, this arrangement both relied upon and reproduced an anti-Black ontology. Reduced to the property of others, Black people were denied the characteristic Locke and his followers deemed fundamental to ‘man’ – having ‘property in his own person’ (p. 181). Following abolition, new mechanisms were instituted to mark the boundaries of the property-owning subject. An important mechanism of boundary making was nuisance law, which was reworked in the late 19th century to organize racial exclusion in two ways: through property-specific restrictions on resale and renting (racial covenants) and neighbourhood-specific restrictions on land uses (zoning). As Freund (2007: 227) explains, these two restrictions sometimes applied exclusively to Black people and sometimes applied to a broader category (e.g. tenants or people of colour) that was deemed ‘a threat to the “health” and “stability” of neighbourhoods’, especially white suburbs. While best documented in the United States, a smaller literature examines nuisance-based racial exclusions, whether racial covenants or zoning, in 20th-century Canada (Allen, 2019; Mathieu, 2010; Peters et al., 2019).

All of these anti-Black socio-spatial relations, of course, are contested by Black people, sometimes through organized resistance (Isoke, 2013) and always through the existential necessity of ‘searching for ways to halt and/or undo the constraints that enclose them in states of unfreedom’ (Mugabo, 2019: 14). Attending to the ‘spatialities of blackness’ is thus ‘a method’ (Hawthorne and Heitz, 2018), a structured way of analysing socio-political life that both ‘disrupts . . . normative conceptualization[s]’ of subjects and spaces (Eaves, 2017: 80) and illuminates more liberatory ‘genres of the human’ (Wynter and McKittrick, 2015). New genres of the human, importantly, emerge alongside new genres of *geography,* geographies that are ‘not tied to already existing analytical cosmogonies that refuse black life’ (McKittrick, 2017: 99). As a white anti-racist scholar, my purpose is less to document or theorize Black people’s redefinitions of the human than to engage Black radical thought to push back against, and work collectively to destroy, the white supremacist and anti-Black discourses and practices that seek to circumscribe, displace or eliminate such redefinitions (Wright et al., forthcoming).

The history of Montreal reveals particular instantiations of the general relationship between the human and the less-than-human, between geography and the ungeographic. Montreal was founded as a French settler colony on Haudenosaunee and Anishinaabeg land in 1642. Like other locations in the French Atlantic, its original relationship to blackness was formed through slavery. The city, though it never enslaved people on the scale of the French colonies in the Caribbean and the southern United States, subjected roughly 518 Africans to enslavement between 1642 and 1834 (Trudel, 2004: 96), a period during which the city’s overall population grew from a few hundred to around 30,000 people. In legal terms, Black enslavement in Montreal and elsewhere in the French Atlantic was codified by the French jurist Jean Bodin (1530–1596). For Bodin, enslavement was justifiable on the condition that it originated from the capture of an enemy combatant in a just war. This provision effectively rationalized the trading of enslaved Africans (imagined to have been produced by incessant wars in the African interior) while barring the enslavement of French people (Rushforth, 2012).

Geographically, Bodin’s justification for slavery constituted multiple scales of Black exclusion. At the largest scale, Bodin provided a historically novel conception of territory. As Elden (2013) explains, Bodin was the first thinker to link sovereign power to the control of a delimited territory, rather than simply control over people and resources. This fashioning of territory, however, was an accessory to enslavement, as it was Black people’s supposed capture by warring nations in another territory that justified their (continued) enslavement in French territory (Rushforth, 2012). As Bodin spatialized sovereign power at the scale of the territory, he also drew limits around its exercise. Property was one such limit. Property, Bodin argued, belonged to the household, and only ‘despots and tyrants’ interfered in its disposition (Bodin, 1993 [1583]: 509–510). The household, it was assumed, functioned as a miniature state, with the adult male regarded as the legal owner of property, including the enslaved. In this move, Bodin codified property in land and people (the enslaved), placing both under the rule of the father and the home rather than the sovereign and the territory.

Bodin’s thought, this suggests, articulates a particular racial regime of ownership, configuring the potential property owner in the French colonies in racial (as well as gendered) terms. The property owner, for Bodin, is a male citizen. Such a person may or may not actually own property, and the profound inequalities of French colonial society ensured that many did not (see Greer, 1997). Even potential property ownership, however, was barred to the enslaved. The latter were non-citizens, and doubly so. Bound to the rule of the father and the home, the enslaved had no place in the community of citizens and, thus, no property the sovereign (or other citizens) was bound to respect. Supposedly captured in war, moreover, the enslaved had ceased to be members of any *other* community of citizens, as anything ‘won in war is the property of the victor’ (Bodin,1993 [1583]: 82). The enslaved, as scholars have argued in other contexts, inhabited a kind of socio-spatial nowhere, evicted from any space in which they might become citizens or property owners (cf. Da Silva, 2007; Hartman, 1997). The proper space of enslaved Black life here is neither public space nor private space, neither domestic territory nor foreign, but ‘a profoundly disturbing nowhere’ (McKittrick, 2017: 98).

This configuration of racial subjects and racialized spaces carries into present-day Montreal in a variety of ways. As elsewhere in the Americas, the clearest line of continuity passes from the category ‘slave’ to the category ‘criminal’. As Nelson (2016) explains, enslaved Black people in Montreal were always ‘hyper-visible’ due to their small numbers, and an informal regime of white, criminalizing surveillance tracked their movements in public space. As the cause of abolition gained support in the late 19th century, moreover, Montreal slave owners mobilized the spectre of criminality to preserve their human property. ‘We are all very convinced’, a group of slave owners told the colonial government in 1800, ‘that this class of men [sic] . . . live an idle and abandoned life and could be tempted to commit crimes, of which it is the duty of all good citizens to try to prevent’ (Trudel, 2004: 251). The inclusion of Black people within the bounds of citizenship, in this context, was an impossibility. A Black person could be a non-citizen (a slave) or a criminal, nothing more. We have a glimpse, here, of the passage from blackness as a signifier of slavery to a signifier of criminality that, as I noted above, has been documented in the United States and elsewhere in the Americas. My task in this article is not to retrace a centuries-long history, but to show how an early association between blackness and criminality in Montreal was rearticulated in the 1980s amid important, tenant-favouring changes in property relations.

My analysis in what follows draws on a mix of methods. It draws primarily on archival research, including the fonds of the Montreal police department, the municipal housing authority, a para-municipal housing developer and two community organizations. It also draws on court records and a close review of the print and television media. It draws, finally, on 19 semi-structured interviews with city councillors and city employees who worked on housing issues in this period and two interviews, conducted for a larger project, with Black tenants who were criminalized and evicted. My approach to these materials is genealogical, seeking to show how new social and spatial categories emerge within subtly changing relations of power – categories, in particular, that construct the racial boundaries of the tenant.

## Tenants’ rights and the meshwork of property

The tenant occupies an ambiguous position in contemporary property relations. Making sense of this position requires that we reject from the start the standard ‘ownership model’ of property, a model that imagines a strict inside/outside relation to property: one is either an owner or a non-owner (Blomley, 2004). Tenants and tenants’ rights, in this model, are difficult to locate. As Blomley (2020: 4) explains, property is better understood as ‘a relational meshwork’ in which ‘we are all variously positioned’. In this meshwork, a tenant has a more precarious relationship to property than an owner but is still closer to the position of an owner (more secure) than someone who is neither an owner nor a tenant. This meshwork, as Blomley emphasizes, is necessarily a venue of socio-political struggle, in which relative positions of privilege and precarity are liable to contort and change. The latter, I will suggest, is precisely what happened in 1980s Montreal, as tenant groups won conditional recognition of the ‘right to remain’ in their rented homes.

The relevant positions in this meshwork, of course, are not simply ‘landlord’ and ‘tenant’. Or rather, as Blomley recognizes, a tenant is never *just* a tenant, and other aspects of a tenant’s social position, including their racial background, can greatly affect their relationship to property. Racial covenants, for example, prohibited owners from selling or renting to people of colour, while land-use zoning could prevent the construction of new rental housing (with racial implications that are well documented). Housing inspections, ostensibly meant to ensure decent living conditions, have been used to surveil and criminalize Black tenants (Hartman, 2019; Rodriguez, 2021). People of colour face also systemic discrimination from residential landlords (Feagin, 1999; Lipsitz, 1995; Massey and Denton, 1993), while Black and Latino tenants are displaced at disproportionate rates (Desmond, 2012; Hartman and Robinson, 2003).

Much of the recent literature on racialized tenant evictions has focused on variations of the United States ‘one strike’ law (see Delaney, 2004; Fagan et al., 2012). The latter originated in Bill Clinton’s Housing Opportunities Program Extension Act of 1996 and was validated by the Supreme Court in *HUD v. Rucker* (2002). The law permits public housing authorities to evict a tenant on the suspicion that they, or a member of their household, have committed a criminal act. In practice, as Tiffany Lethabo King (2010) explains, the law disproportionately targets Black households. In so doing, the law enlists and perpetuates the longstanding social and spatial logics of anti-blackness that position Black people outside the sphere of socio-spatial belonging. Underlining the specific precariousness of Black tenants, King argues that one-strike evictions merge the centuries-old perception of Black people as ‘ungeographic’ and the intensifying criminalization of Black life in the post-civil rights era into ‘evict-able bodies’ (p. 55). Anti-blackness, King suggests, shapes the relational meshwork of property, producing a precariousness specific to Black tenants.

These insights help to make sense of the apparent gains of tenant groups in 1980s Montreal. These gains can be traced back to the 1960s, when a series of housing committees were formed in white working-class neighbourhoods in the province of Quebec and began to forge alliances with labour unions and left-wing political parties (Favreau, 1989; Regroupement des comités logement et associations de locataires du Québec (RCLALQ), 2018; Saillant, 2018). At the provincial scale, these actors achieved a major advance with the election of the left-wing, sovereignist Parti Québécois (PQ) in 1976. One of the PQ’s early moves once gaining power was to undertake a major study of tenant–landlord relations in the province, the *Livre blanc sur les relations entre locateurs et locataires.* The report, though it fell short of many activists’ expectations, became the first provincial document to recognize that tenants, not just property owners, had an essential right to remain in their homes on a ‘stable and continuous’ basis. ‘Housing’, the report explained, ‘is essential to the maintenance of one’s life’ (Québec, Gouvernement du, 1977: 5). It was important, therefore, that tenants be accorded the same ability to ‘maintain their life’ as property owners. ‘In a traditional society, property rights often appear as absolute rights’, the report explained. ‘However, in a country where the majority of the population are tenants, the right to remain in one’s home (as a tenant) needs to be considered equally important’ (Québec, Gouvernement du, 1977: 21).

The conception of property in the report is less juridical than biopolitical (see Rutland, 2015). Property, in the form of housing, may legally belong to individual owners, but it is also essential to the life of non-owning individuals and the population in general. It follows from this view that juridical rights to property need to be reworked: a new right, ‘the right to remain’, needs to be recognized alongside more ‘traditional’ property rights. The recognition of this new right in the *Livre blanc* soon became the basis of several important legislative changes in Quebec, including the establishment of a landlord–tenant tribunal (1979) and major revisions to the Quebec civil code (1980). The latter included the addition of an article stating that ‘all (residential) tenants have a personal right to remain in their home’. This right to remain, although it could be abrogated under certain conditions, represented an important shift in the ‘relational meshwork’ of property. Tenants, here, acquired more security of tenure. This new right, established in 1980, has been regarded by housing activists as the ‘cornerstone of the right to housing’ ever since (RCLALQ, 2015).

At the municipal level, tenant groups also made gains in the 1980s. The first achievement came in 1984, when the city adopted a new and much tougher housing code. The code gave the city new powers to act against landlords who allowed unsanitary, unsafe or nuisance conditions to persist in a housing unit or building. The second achievement came during the 1986–1994 mandate of the Rassemblement des Citoyens de Montréal (RCM), a left-wing municipal party formed in 1973 and elected to a majority in 1986 (Leveillée, 1988). The RCM’s broadest housing policy, *Habiter Montréal*, included commitments to improve the quality of the residential environment, enable access to property ownership, expand social housing opportunities and ‘support the maintenance of people in their milieu’ (Montréal Ville D, 1989: 4). Like the PQ’s *Livre blanc*, the RCM’s policy saw the right to remain in their homes as an essential condition of life – for tenants, as well as property owners. ‘The attachment of Montrealers to their residential environment is great’, the policy explained. ‘It is at the centre of community life, and people’s familiarity with this environment is a contributor to quality of life and security’ (Montréal, Ville de, 1989). Property, here again, acquires a biopolitical character, an essential relationship to the life of individuals and communities.

Together, these changes reworked the position of the tenant in the relational meshwork of property. A strong tenants’ movement based in white working-class neighbourhoods and the election of progressive governments at the provincial and municipal levels freighted new ideas about the biopolitical connection between tenants and the residential environment. Guided by these ideas, a series of legal, policy and program changes brought greater attention to tenant living conditions and the recognition of a new tenant right, the right to remain in one’s rented home. While these changes seem to bring the tenant somewhat closer to the owner position in the meshwork of property, their racial implications require closer attention, especially as new forms of Black criminalization emerged in the same period.

## The criminalization of blackness

New forms of Black criminalization began to emerge in the late 1970s, as Montreal’s Black population experienced a major expansion. This expansion was enabled by changes in Canadian immigration policy in the 1960s, changes that allowed entry to non-white populations after decades of an effective ‘White Canada’ policy (Taylor, 1991). Following these changes, new flows of Black migration helped to lift Montreal’s Black population from 15,000 in 1971 to 101,390 in 1991 (Torczyner & Springer, 2001, 16; Williams, 1997, 162). The city’s overall population grew more modestly in this period, increasing from 2,677,620 to 3,076,160 (Ornstein, 2007). By the 1980s, Black migration had begun to produce a clear settlement pattern, a pattern that partly reflected the city’s longstanding linguistic divide: French speakers on the east side of the city, English speakers in the west (Mugabo, 2019). Haitian migrants, either French speakers or apt to learn the language, tended to settle in the northeast, especially the neighbourhoods of Saint-Michel and Montréal-Nord. Jamaicans and other anglophone-Caribbean migrants tended to settle in the west, especially Little Burgundy (the longtime heart of Montreal’s Black community), Côtes-des-Neiges, Notre-Dame-de-Grâce and Cartierville (Williams, 1997).

As the city’s Black population increased, so did the evidence of anti-Black racism in various forms (Mugabo, 2016; Williams, 1997). Anti-Black policing was a particular problem. Tellingly, Black activists increasingly noted that the police, when called by a Black person, often arrested the Black victim of the crime rather than the (usually white) perpetrator (Negro Community Centre (NCC), 1979). In the eyes of the law, a Black person could not be a citizen, only a criminal. Woven through anti-Black policing, moreover, was a geographical displacement reminiscent of Bodin’s thought. The police, for example, frequently demanded to see the immigration papers of Black residents, sometimes detaining them until their immigration status could be verified. The police, as one Black organization noted in 1979, tended to assume ‘that all Blacks are immigrants’ and generally ‘in Canada illegally’ (NCC, 1979: 2). In the same year, the police descended on a group of Haitian-Montreal youth playing soccer – noisily, white neighbours claimed – in an east-side park. The police, after insulting, threatening and beating several of the youth, announced the arrested youth would be taken to Fort Dimanche, a Haitian prison built under US occupation that became notorious for torture under the Duvalier regimes (Leclerc, 1979).

Black criminalization and discursive displacement intensified in the 1980s amid increasing (white) concerns about concentrated, or ghettoized, Black life. At the centre of these concerns were demographic data revealing a declining white birthrate in Quebec alongside increasing non-white immigration and reproduction. For many white commentators, these developments signalled a long-term decline in white demographic and cultural dominance and the possible ‘disappearance’ of the Québécois (people of French ancestry in Quebec) as a nation (see Mercier, 1989). All of this put increased attention on non-white assimilation into the white majority culture and marked spaces of significant Black residence as unassimilated ‘ghettos’. Not surprisingly, these references to Black ‘ghettos’ often included claims about danger and criminality. In an exemplary article in *La Presse,* a journalist reflected on a recent visit to Côte-des-Neiges, during which he observed congregated Jamaicans. ‘For the first time in my life’, he wrote, ‘I felt that Montreal could soon contain a restricted zone, a neighbourhood where one could not venture without hesitation’ (Leblanc, 1988). This situation, for the journalist, needed to be addressed. The ‘ghettos’ that had recently come into existence needed to be ‘broken’.

By the late 1980s, references to Black ‘ghettos’ became commonplace, especially as the police began a mediatized campaign against supposed Black gang members and drug dealers (see Rutland, 2021). These areas, to be clear, bore little resemblance to the racially segregated Black neighbourhoods of some US cities. In Montreal, Black people have never accounted for even *half* the population in any census tract and have only topped a quarter of the population in a small number of tracts. As in Britain and France, however, the spectre of lawless US ghettos served to ignite a moral panic over Black spatial practices in Montreal and licensed a massive deployment of police personnel and weaponry (Hall et al., 1978). A major police operation against Black street-level drug dealers in the west-side neighbourhood of Cartierville, for example, was described by the media as an attack on the ‘Bronx of Crack’ (Rancourt, 1989; Saint-Jean, 1989). A similar operation in Notre-Dame-de-Grâce 18 months later was described as targeting ‘a little Harlem’ (Rancourt, 1991). In these and other cases, the imagined elsewhere of US cities – places imagined to be nothing but hellscapes of drugs and violence – sutured the perceived connection between concentrated blackness and criminality.

These references to US ghettos, more than just criminalizing the targeted areas and their residents, also painted the latter as something foreign, something that had entered illegitimately into the territory and population of Montreal. Although Black people had resided in Montreal for centuries by this point, there was apparently no *local* spatial referent for areas of significant Black residence. Even Little Burgundy, the longstanding heart of the Montreal Black community, was frequently described as ‘Little Los Angeles’ by the police and social workers in this period (Anonymous, 2017). These references mirror Mugabo’s (2016) claim that blackness in Quebec is a conceptual ‘impossibility’ while signalling a continuation of the historical developments I traced above. As in the 17th and 18th centuries, the proper space of Black life was located outside the territory of the nation. It was located in an imagined elsewhere that had somehow illegitimately found its way inside. This spatial contortion, an updated version of a longstanding discursive displacement, raises questions about the new tenants’ right acquired in this same period. To what extent can Black tenants claim a right to remain in their homes when their belonging in the territory is nullified in advance? An answer to this question can be bound by looking at the two most important housing interventions of the progressive RCM city government.

## The evicted space of Black tenants’ rights

The first important housing intervention was the RCM’s signature *Programme d’acquisition de logements locatif* (PALL). Launched in 1989, PALL saw the city buy up dilapidated rental housing units, renovate them and then sell them off to the city housing authority, a non-profit organization or a tenants’ cooperative. Through the program, over 3000 housing units were purchased, renovated and converted into social housing between 1989 and 1994. The program was about more than housing units, however. The goal was also to revitalize entire neighbourhoods, creating a ‘sense of attachment’ between residents and their milieu (Société d’habitation et de développement de Montréal (SHDM), 1993a: 3). As this implies, the sense of attachment described in the RCM’s *Habiter Montréal* policy did not exist in all neighbourhoods. It was missing in spaces RCM and PALL officials consistently called ‘ghettos’, spaces that interrupted the biopolitical connection between residents and their milieu and that, if allowed to exist, were liable to expand. As a PALL report explained, ‘the philosophy [of this program] is to “break” the cycle of deterioration in certain sectors . . . and prevent the creation of ghettos, in which social problems (criminality, etc.) concentrate’ (SHDM, 1993a: 7).

Not surprisingly, the program focused disproportionately on neighbourhoods of significant Black residence. Three such neighbourhoods – Côte-des-Neiges, Notre-Dame-de-Grâce and Cartierville – represented 54% of interventions (SHDM, 1993b). These neighbourhoods, already described as ghettos by police, were given new attention by housing officials and media reports on the PALL program. Here, details about criminality were paired with descriptions of physical conditions, mirroring the form of ‘slum surveys’ in the reform and urban renewal eras (Hartman, 2019; Rutland, 2018). In an article titled ‘City hopes housing project will save street’, the *Montreal Gazette* described a sector of Côte-des-Neiges targeted by PALL as one of the city’s ‘bleakest’:

The sidewalks are cracked, graffiti is scrawled across red-brick buildings, and most front lawns lie eroded from lack of attention. Many apartment buildings stand boarded up after years of neglect by absentee landlords. A few buildings were abandoned by tenants after crack dealers invaded the apartments. (Derfel, 1991)

Descriptions of drug dealers and sex workers ‘invading’ an area were common in PALL and media reports on the housing program, suggesting that something foreign had entered the city and taken over from local residents. Again, ‘the Bronx’ was a common signifier of such foreignness. For *La Presse,* the PALL program succeeded in eliminating several ‘Little Broxes’ (Laberge, 1995) around the city while also preventing some from appearing in the first place ‘by intervening as soon as the shadow of the Bronx appeared’ in an area (Gruda, 1995).

While it was often suggested by the media and PALL reports that criminality was eliminated through renovations and other physical improvements (Bernèche, 1997; Montreal Gazette, 1994), a variety of social interventions were also made. In some cases, suspected drug dealers were evicted immediately after the city’s purchase of the building. In other cases, the city arranged with the police to keep the buildings under surveillance. An examination of court records shows that police interventions in PALL-targeted buildings decreased but remained high following their acquisition by the program. An average of 136 building residents per year were arrested and charged in the three years before the acquisition, 103 in the year of acquisition and 46 per year in the three years after the acquisition. In addition to evictions and police interventions, tenants were also displaced by indirect pressure, including the installation of video cameras, an intercom system an armed security guard (in at least three cases), and the simple fact of becoming tenants of the state rather than an absentee landlord.

The consequences of the PALL program for Montreal tenants are a matter of perspective, which is to say, a matter of social position. For many tenants, the program created better living conditions (through the renovations) and greater protection from displacement (through the creation of social housing). PALL reports and media stories emphasized these gains, often providing dramatic before and after images of PALL-targeted neighbourhoods PALL reports and media stories emphasized these gains, often providing dramatic before and after images of PALL-targeted neighbourhoods. The ‘before’ images included dramatic depictions of criminality, violence and fear. The ‘after’ images centred *un*dramatic – because normative – depictions of household and neighbourhood life. These changes, however, were achieved through significant direct and indirect displacement, the elimination of criminalized Black tenants and anyone who preferred not to live under state surveillance. Their tenant rights, including their right to remain, were foreclosed in advance, as their potential attachment to the neighbourhood was nullified, depicted as foreign and deemed detrimental to the environment of others. Their presence turned an environment (to which one could be attached) into a ghetto. ‘The tenant’ whose rights were protected by PALL was thus a racial subject, a figure constituted as the edges of criminalized blackness.

While PALL was a high-profile program, the second major housing intervention – the eviction of suspected drug dealers – proceeded more quietly. The evictions, begun in 1989, targeted both public and private housing and employed different mechanisms in each setting. Evictions in public housing centred on the largest housing complex in a Black neighbourhood, *Ilots Saint-Martin* in Little Burgundy. In the fall of 1989, as the activities of low-level Black drug dealers were attracting the attention of the media and the police, RCM mayor Jean Doré called a meeting of city officials, police officers and public housing officials and devised a plan of attack. Part of the plan was a brutal police operation that endured for at least four years. Another part called for the public housing authority to assist the police by providing the floor plans of the housing complex, the keys to its entryways and eventually the master key that opened the door to all housing units (Anonymous, 2017). The final part of the plan called on the housing authority to evict tenants suspected of selling drugs themselves or allowing their unit to be used for such purposes. Through this action, roughly 60 households were evicted from *Ilots Saint-Martin* in 1989–1991, the vacated units kept empty for years thereafter to combat drug dealing (Goyette, 2017).

The use of evictions to combat drug dealing began in the United States in the mid-1980s (Mazerolle and Roehl, 1998; Weed, 1997). Termed a ‘civil remedy’, evictions have been useful because of the lower standard of evidence they require: a criminal activity must be proven ‘beyond reasonable doubt’, while civil violations are based on a lesser standard sometimes called ‘clear and convincing’ evidence in the United States (Cheh, 1991) and the ‘balance of probabilities’ in Canada (Gallant, 2014). As King (2010) emphasizes, evictions also made it possible to punish entire families for the probable activities of a single person. In Canada, the Toronto public housing authority began to employ this measure in 1988, evicting 89 households in that year (Malarek, 1989). The evictions relied on a provision of the Ontario Landlord and Tenant Act, which allowed the removal of a tenant when he or she ‘exercises or carries on, or permits to be exercised or carried on . . . any illegal act’ (Malarek, 1989: 13). The phrase ‘permits to be carried on’ was essential, as entire households could be evicted on ‘probable’ evidence that a single person, who may or may not have lived in the housing unit, sold drugs there.

In Montreal, a more general provision of landlord–tenant law enabled public housing evictions. The provision, article 1618 of the Quebec civil code, prohibited the tenant from changing the ‘form or destination’ of the rented property. The article was added to the civil code in 1973 but did not apply to public housing until 1979, when the aforementioned efforts of the PQ to strengthen tenants’ rights finally subjected public housing authorities to the tenant protections of the civil code (Goyette, 2017; Stanhope and Hamel, 1980). This change, premised on protecting tenants’ rights, ultimately became the means of evicting Black tenants and their families by scripting drug dealing as a necessarily commercial activity (and thus, a change in the ‘destination’ of the unit). Drug dealing, whether undertaken by the tenant or someone visiting the housing unit, was seen as transforming the unit into something other than a rented home, something other than a ‘milieu’ upon which the tenant’s life depends. Something external, non-residential, thus interrupted a residential space and the rights that prevailed within it.

In practice, the distinction between residential and commercial was often a distinction between different *kinds* of residential life. In one eviction case, considered a precedent for the application of article 1618 to drug dealing, the housing authority alleged a Black mother in Little Burgundy was ‘not using the unit for residential purposes’. The evidence, provided by a police officer, was that people were regularly observed ‘coming and going’ from her unit and that, during a raid, crack had been seized from a person in her unit. The mother could not refute the claims. For years, she had welcomed community members to her home. ‘People would come over all the time’, she recalls. ‘I would cook for them, take care of the children. People would play cards, drink, and sometimes smoke pot’ (Alleyne, 2020). When crack entered the community in 1988, it changed some of her friends’ activities. While she discouraged people from smoking crack, she did not bar them from her home. ‘The people who smoked crack, I knew them before they started’, she recalls. ‘They continued to come over. Sometimes, they just needed a place to come down from a high, so they’d come over and chill’. Here, then, a non-normative use of space, providing a second home to poor Black adults and children, was deemed non-residential. Allowing her home to be used this way cast her and her children as non-tenants – ‘evict-able bodies’, in King’s phrasing.

In private housing, the eviction of suspected drug dealers relied on a different mechanism. The municipal housing code, passed in 1984 to ensure safe and sanitary conditions in rental housing, included an article defining a ‘nuisance’. Adopting the customary legal form, the code defined a nuisance as any space that, ‘because of the use made of it or the conditions in which it is, is such a nature as to be detrimental to the health, safety, or welfare of the residents or of the public’ (Montréal Ville D, 1984). Any space found to be a nuisance could be designated ‘unfit for housing’ by the city and barricaded until the landlord addressed the offending conditions. In 1989, as the police and public housing authority began their war on supposed Black drug dealers, the code’s protection against nuisance conditions in private rental housing was turned against criminalized Black tenants. The move, though welcomed by the police, was initiated by the RCM-headed municipal housing committee. ‘We realized we could use the housing code to eliminate drug operations’, remembers an RCM councillor and member of the committee. ‘It was a more efficient intervention than the police because the police need criminal evidence, but we only needed to show there’s a nuisance. It was as simple as that’ (Bennett, 2018).

In fact, housing inspectors worked closely with the police. In advance of a drug raid, the police alerted city housing inspectors. Once the raid was completed and the suspects arrested, the police called housing inspectors to barricade the unit and issue a notice of evacuation to the landlord. In many cases, the inspectors also pinned a poster to the barricaded unit, announcing that it was the home of a drug dealer. As the notice of evacuation explained, the city would remove the barricade only after the landlord had legally evicted the arrested tenant. Although the involvement of the police was essential, the RCM councillor’s claim about the efficiency of the process is correct. Criminal evidence was still required to convict a suspected drug dealer, but barricading an apartment simply required the police to tell the housing inspectors that the unit was used to sell drugs. A suspect could later be found innocent, or be released on bail pending their trial, and find themselves barred from their home. Appreciating the efficiency of the process, the police and housing inspectors used this method to evict at least 21 households in 1990 (Service de police de la Communauté urbaine de Montréal, 1990). Numbers thereafter are unavailable, but media coverage shows the method continued to be used in 1991 and 1992 and focused predominantly on Côte-des-Neiges, Notre-Dame-de-Grâce, and Cartierville.

As I discussed above, using nuisance provisions to control Black tenancy has a long history in North America. While certain measures, such as racial covenants, have been explicit in their racial exclusion, others have been more subtle. Nuisances, for example, have historically been defined on the basis of behavioural norms that, as (Godsil, 2006) explains, are modelled on whiteness. Many US state laws were also amended in the 1990s to define drug dealing as a nuisance, with all the racial implications of the broader war on drugs (Weed, 1997). The nuisance provision used in Montreal was general in orientation. Neither the article of the housing code nor the notice of evacuation issued to non-compliant landlords mentioned race or drugs. Its practical usage, however, relied on implicitly racist behavioural norms. In the only public defence of the measure, an RCM councillor explained that ‘the city has long possessed certain powers and has decided to use them to aid the police in the context we know’ (Boisvert, 1992). The implied context was the police’s war on drugs in the city’s ‘ghetto’ neighbourhoods, places that the police, media and city officials had defined as socially and spatially aberrant – places foreign to the ‘residential environments’ that the RCM believed contributed ‘to quality of life and security’.

Black tenants no doubt resisted these housing interventions in various ways, most of them invisible to the archives, the media and the courts. A particularly important example involved an Afro-Dominican mother whose rented apartment in Petite-Patrie was raided by the police in 1992. Although she was arrested on drug charges, no drugs were found in her home, and she was released the following day. Her home, however, was barricaded by city housing inspectors under the nuisance provision discussed above, forcing her and her six children to stay at a friend’s home. The mother ultimately brought the city to court, challenging the barricading of her home and the city’s wide interpretation of the nuisance provision (*Perez v. Ville de Montréal,* 1992). The case stressed the (in)constitutionality of the city’s action, its contravention of the Canadian Charter of Rights and Freedom, but also subtly challenged the period’s dominant representation of Black homes and neighbourhoods. In her affidavit, the mother detailed her attachment to various neighbourhood spaces, especially the schools her children attended on foot each day. It also described the materials of domestic life concealed by the city-imposed barricades, including children’s toys, a piano and gymnastic equipment. She and her six children, she explained, were deprived of their constitutional rights and their right ‘to the peaceful enjoyment of our belongings’ (*Perez v. Ville de Montréal*, 1992: 3).

The more mundane materials of her family’s domestic life were invoked in a different way. Submitted to the court were a series of photos taken by the mother, mostly photos depicting her children asleep in their exiled environment (a friend’s apartment). The children, huddled together under a thin blanket on the floor, conjure a biopolitical attachment to one’s milieu in the negative, a *prior* attachment pulled apart by their eviction. More than anything, the photos depict the vulnerability of the body and the conditions it depends upon – precisely the biopolitical attachment 1980s housing policy recognized in general, but ignored in the city’s ‘ghettos’. The photos also suggest that ghetto conditions, if they exist at all, are actually produced by efforts to eliminate them. They are created not by Black people but by the racial state. The city, in the end, never bothered to defend its action, and the court ordered that the city’s barricades be removed 25 days after they had been erected. The court’s ruling in the mother’s favour also brought an end to the use of the housing code to evict criminalized Black tenants, an important victory.

## Conclusion

If property relations construct racial subjects, then it essential to understand the meshwork in which this occurs. Guided by recent work on property and Black geographies, this article examined how racial subjects were formed in struggles over tenants’ rights in 1980s Montreal, ultimately constituting a better-protected ‘tenant’ at the edges of blackness. This result, I showed, is traceable to the ontological and geographical displacements of slavery: the eviction of Black people from the category of the human, as well as the categories of territory and property. This regime of property and racial subjects, originating in French Atlantic slavery, survived the abolition of slavery primarily through the criminalization of Black life. This can be observed in struggles over housing in the 1980s, when new housing institutions, policies and programs pushed by white tenant activists and established by progressive governments to protect tenants’ rights became the very mechanism through which Black tenants were evicted. This racial lacuna in progressive politics and tenants’ rights discourse was rooted in a broader structure of power. Black tenants’ right to remain in their homes, in this context, was subverted by the longstanding and ever-evolving conception of Black people as ungeographic and the relegation of their proper place to a distant, imaginary and ‘profoundly disturbing nowhere’ (McKittrick, 2017: 98).

Like other subjects of property relations, the constitution of the tenant occurs within evolving racial regimes. The tenant, as Blomley (2020) has shown, occupies a middling position in the meshwork of property: more precarious than a property owner, but less precarious than someone who is neither an owner nor a tenant. The tenant, however, is never just a tenant, but also a racial subject. In Montreal, the ‘tenant’ whose rights and living conditions were improved by political developments in the 1980s was something other than a universal, race-neutral subject. It was a subject whose rights were threatened only by a landlord, a subject whose rights were not foreclosed in advance by a negated right to exist within the territory in the first place. The tenant, in sum, is a subject who lived in an ‘environment’ or ‘milieu’, not a ‘ghetto’, a subject whose decreased precarity in the meshwork of property was ultimately produced alongside and through increased precarity for Black tenants. The tenant, in other words, is a figure who can appear and whose rights can be protected in a space cleared of criminalized Black tenants and the ‘ghetto’ conditions they purportedly create. Tenants’ rights appear in the evicted space of Black tenant rights.

If Black people, while being excluded from the category of the human, also produce new genres of the human (Wynter and McKittrick, 2015), then Black tenants also produce new genres of the tenant. Resistance to racist evictions and spatial controls occurs in various ways. At times, legal remedy is sought by seeking inclusion within normative categories. As in Petite-Patrie, an evicted Black mother can point to her family’s attachments to the neighbourhood and their biopolitical dependence on their home. A Black tenant, she claims, is still a tenant. Her home and neighbourhood are still a milieu. At times, however, resistance also calls normative categories into question, challenging the abstract ideas and socio-legal institutions that configure the socio-spatial boundaries of tenancy. As in Little Burgundy, a Black mother can open her home to the community and provide a space of support and respite in the midst of a drug-related health crisis and a state-engineered social crisis. In the eyes of the landlord–tenant tribunal, this use of space defied the normative category of ‘residential’ – and indeed it did. In such defiance, however, we find other genres of the tenant, other ways of organizing relations to each other and space, and a potential basis of tenant struggle that is not simply more racially inclusive but also more disturbing to the property relations that constitute tenants in the first place.

## Note

1 All translations from French language sources are my own.
